# Supplementary material for: Invisible noise obscures visible signal in insect motion detection
Source: Sci Rep. 2017 Jun 14;7:3496. doi: 10.1038/s41598-017-03732-7 (PMC5471215; doi:10.1038/s41598-017-03732-7)
Supplement: Supplementary file 1 — Supplementary Information [file 41598_2017_3732_MOESM1_ESM.pdf]

Supplementary Information

Paper: "Invisible noise obscures visible signal in insect motion detection". Authors: "Ghaith Tarawneh, Vivek Nityananda, Ronny Rosner, Steven Errington, William Herbert, Bruce G. Cumming, Jenny C. A. Read and Ignacio Serrano-Pedraza"

Spatio-temporal Filters

Human simulations

The spatial filters were second and third derivatives of Gaussians:

$$SF_1(x) = \frac{(\sigma^2 - x^2)}{\sigma^4} \exp\left(\frac{-x^2}{2\sigma^2}\right) \tag{25}$$

$$SF_2(x) = \frac{(3x\sigma^2 - x^3)}{\sigma^6} \exp\left(\frac{-x^2}{2\sigma^2}\right) \tag{26}$$

with $\sigma = 0.08°$. The temporal filters were

$$TF(t;n,k) = (kt)^n \exp(-kt)\left(1/n! - (kt)^2/(n+2)!\right) \tag{27}$$

with $n = 3$ for TF_1, $n = 5$ for TF_2 and $k = 105$ for both filters (10). These filters are approximately in quadrature.

Mantis simulations

The spatial filters were Gaussian:

$$SF_1(x) = \exp\left(\frac{-(x - \Delta x/2)^2}{2\sigma^2}\right) \tag{28}$$

$$SF_2(x) = \exp\left(\frac{-(x + \Delta x/2)^2}{2\sigma^2}\right) \tag{29}$$

where the separation between filters was $\Delta x = 4°$ and $\sigma = 2.56°$ (corresponding to an acceptance angle of 6° for ommatidia). The temporal filters were first-order low/high pass filters:

$$TF_1(t) = \exp(-t/\tau_L) \tag{30}$$

$$TF_2(t) = \delta(t) - \exp(-t/\tau_H) \tag{31}$$

with τ_L = 13 ms, τ_H = 40 ms. The Laplace transforms of these temporal filters are:

$$\mathscr{L}(TF_1) = (s + \tau_L^{-1})^{-1} \tag{32}$$

$$\mathscr{L}(TF_2) = 1 - (s + \tau_H^{-1})^{-1} \tag{33}$$

The impulse responses of all filters used in simulations (both mantis and human) are plotted in Figure 8.

Mathematical Details of the Derivations

For completeness, we here add some details of the mathematical derivations which were omitted from the main text for brevity.

Derivation of opponent energy

To derive Equation 9, we stated that we substituted for the filter outputs in Equation 8 and simplified. This works as follows. Substituting Equations 2 - 5 into Equation 8:

$$\begin{aligned} \text{Opponent Energy}(t) =& C^2 G_{S1} G_{S2} G_{T1} G_{T2} \\ & \times \Big(\sin\left(2\pi f_T t + \beta + \phi_{S2} + \phi_{T2}\right) \sin\left(2\pi f_T t + \beta + \phi_{S1} + \phi_{T1}\right) \\ & \quad - \sin\left(2\pi f_T t + \beta + \phi_{S2} + \phi_{T1}\right) \sin\left(2\pi f_T t + \beta + \phi_{S1} + \phi_{T2}\right) \Big) \end{aligned} \tag{34}$$

Using the trigonometric identity that $2\sin(A)\sin(B) \equiv \cos(A-B) - \cos(A+B)$,

$$\begin{aligned}\text{Opponent Energy}(t) =& 0.5 \times C^2 G_{S1} G_{S2} G_{T1} G_{T2} \\ & \times \Big(\cos\left(\phi_{S2} - \phi_{S1} + \phi_{T2} - \phi_{T1}\right) - \cos\left(4\pi f_T t + 2\beta + \phi_{S1} + \phi_{S2} + \phi_{T1} + \phi_{T2}\right) \\ & - \cos\left(\phi_{S2} - \phi_{S1} + \phi_{T1} - \phi_{T2}\right) + \cos\left(4\pi f_T t + 2\beta + \phi_{S1} + \phi_{S2} + \phi_{T1} + \phi_{T2}\right)\Big)\end{aligned} \tag{35}$$

and so

$$\begin{aligned}\text{Opponent Energy}(t) =& 0.5 \times C^2 G_{S1} G_{S2} G_{T1} G_{T2} \\ & \times \Big(\cos\left(\phi_{S2} - \phi_{S1} + \phi_{T2} - \phi_{T1}\right) - \cos\left(\phi_{S2} - \phi_{S1} + \phi_{T1} - \phi_{T2}\right)\Big)\end{aligned} \tag{36}$$

Then using the trigonometric identity that $\cos(A) - \cos(B) \equiv 2\sin((B+A)/2)\sin((B-A)/2)$,

$$\text{Opponent Energy}(t) = C^2 G_{S1} G_{S2} G_{T1} G_{T2} \sin\left(\phi_{S2} - \phi_{S1}\right) \sin\left(\phi_{T1} - \phi_{T2}\right) \tag{37}$$

as in Equation 9. Notice that the right-hand side of this equation has no dependence on time, i.e. for a drifting grating, the output of an opponent energy model is constant and does not depend on the grating phase.

When one spatial filter is a shifted version of the other (insect case)

Consider a function $y(x)$ whose Fourier transform is

$$Y(f_S) = \int_{-\infty}^{\infty} exp(2\pi i f_S x) y(x) dx. \tag{38}$$

Now suppose the function is shifted by a distance δ. The Fourier transform of the shifted function,$y(x-\delta)$, is

$$Y_\delta(f_S) = \int_{-\infty}^{\infty} exp(2\pi i f_S x) y(x-\delta) dx = \int_{-\infty}^{\infty} exp(2\pi i f_S (x+\delta)) y(x) dx = exp(2\pi i f_S \delta) Y(f_S). \tag{39}$$

That is, shifting a function by δ introduces a phase shift that is proportional to frequency: the Fourier phase is shifted by $2\pi f_S \delta$ at every frequency f_S.

In the Reichardt detector, the spatial filters are identical but offset in position by a distance Δx. This means that the spatial filters have the same gains at every frequency ($G_{S1} = G_{S2}$ for all f_S in Equations 2 - 5) but their phases are shifted by $2\pi f_S \Delta x$ at each frequency f_S. Therefore, $\phi_{S2}(f_S) = \phi_{S1}(f_S) + 2\pi f_S \Delta x$ in Equations 2 - 5. Substituting this into Equation 9, we see that

$$\text{Reichardt Opponent Energy}(t) = C^2 G_S^2 G_{T1} G_{T2} \sin\left(2\pi f_S \Delta x\right) \sin\left(\phi_{T1} - \phi_{T2}\right) \tag{40}$$

This means that in the Reichardt detector, the opponent energy vanishes as the spatial frequency tends to zero.

When one spatial filter is the derivative of the other (mammalian case)

Now consider the Fourier transform of the function's derivative:

$$Y_1(f_S) = \int_{-\infty}^{\infty} \exp(2\pi i f_S x) \frac{dy}{dx} dx. \tag{41}$$

Using integration by parts, this is

$$Y_1(f_S) = \left[\exp(2\pi i f_S x) y(x)\right]_{-\infty}^{\infty} - 2\pi i f_S \int_{-\infty}^{\infty} \exp(2\pi i f_S x) y(x) dx. \tag{42}$$

Assuming the function $y(x)$ vanishes at $\pm\infty$, this gives

$$Y_1(f_S) = 2\pi f_S Y(f_S) \exp(-i\pi/2). \tag{43}$$

That is, the Fourier transform of the derivative differs from the Fourier transform of the original function, in magnitude by a factor $2\pi f_S$ at frequency f_S, and in phase by a constant $\pi/2$, independent of frequency.

For modelling mammalian motion detectors, we followed (10) in having one spatial filter be the derivative of the other. In terms of Equations 2 - 5, we therefore have $G_{S1}(f_S) = k_S G_{S2}(f_S)$ (where the constant k accounts for the normalisation we apply to the filters), and $\phi_{S2}(f_S) = \phi_{S1}(f_S) + \pi/2$. In (9), the spatial phase term $\sin(\phi_{S2} - \phi_{S1})$ is therefore a constant and the opponent energy is

$$\text{Opponent Energy}(t) = k f_S [G_{S2}(f_S)]^2 G_{T1}(f_t) G_{T2}(f_t) \sin(\phi_{T1}(f_t) - \phi_{T2}(f_t)) \tag{44}$$

where again k is an arbitrary normalisation constant.

For mammalian motion detection, it is also common to use spatial filters which are narrowband Gabor functions differing by $\pi/2$ in phase. For sufficiently narrowband Gabors, it is then approximately true that $G_{S1}(f_S) = G_{S2}(f_S)$ and $\phi_{S2}(f_S) = \phi_{S1}(f_S) - \pi/2$. Once again, then, the spatial phase term in (9) is constant.

Response to a general stimulus

For a sum of gratings (Equation 10) by linearity we simply sum Equations 2 - 5 over the components to obtain

$$A(t) = \sum_{j=1}^{N} A_j \tag{45}$$

$$A'(t) = \sum_{j=1}^{N} A'_j \tag{46}$$

$$B(t) = \sum_{j=1}^{N} B_j \tag{47}$$

$$B'(t) = \sum_{j=1}^{N} B'_j \tag{48}$$

where

$$A_j = C_j G_{S1}(f_{Sj}) G_{T1}(f_{Tj}) \sin\left(2\pi f_{Tj} t + \beta_j + \phi_{S1}(f_{Sj}) + \phi_{T1}(f_{Tj})\right) \tag{49}$$

$$A'_j = C_j G_{S1}(f_{Sj}) G_{T2}(f_{Tj}) \sin\left(2\pi f_{Tj} t + \beta_j + \phi_{S1}(f_{Sj}) + \phi_{T2}(f_{Tj})\right) \tag{50}$$

$$B_j = C_j G_{S2}(f_{Sj}) G_{T1}(f_{Tj}) \sin\left(2\pi f_{Tj} t + \beta_j + \phi_{S2}(f_{Sj}) + \phi_{T1}(f_{Tj})\right) \tag{51}$$

$$B'_j = C_j G_{S2}(f_{Sj}) G_{T2}(f_{Tj}) \sin\left(2\pi f_{Tj} t + \beta_j + \phi_{S2}(f_{Sj}) + \phi_{T2}(f_{Tj})\right). \tag{52}$$

The opponent energy is still $AB' - BA'$, which now gives us

$$\text{Opponent Energy}(t) = \sum_{j=1}^{N} \sum_{k=1}^{N} (A_j B'_k - B_k A'_j) \tag{53}$$

where for any two components j and k:

$$\begin{aligned}(A_j B'_k - B_k A'_j) = {} & G_{S1}(f_{Sj}) G_{S2}(f_{Sk}) G_{T1}(f_{Tj}) G_{T2}(f_{Tk}) C_j C_k \times \Big(\\ & \sin\left(2\pi f_{Tj} t + \beta_j + \phi_{S1}(f_{Sj}) + \phi_{T1}(f_{Tj})\right) \sin\left(2\pi f_{Tk} t + \beta_k + \phi_{S2}(f_{Sk}) + \phi_{T2}(f_{Tk})\right) \\ & - \sin\left(2\pi f_{Tk} t + \beta_k + \phi_{S2}(f_{Sk}) + \phi_{T1}(f_{Tk})\right) \sin\left(2\pi f_{Tj} t + \beta_j + \phi_{S1}(f_{Sj}) + \phi_{T2}(f_{Tj})\right)\Big).\end{aligned} \tag{54}$$

Using the trigonometric identity that $2\sin(A)\sin(B) \equiv \cos(A-B) - \cos(A+B)$,

$$
\begin{aligned}
(A_jB'_k - B_kA'_j) =& 0.5\, G_{S1}(f_{Sj})G_{S2}(f_{Sk})G_{T1}(f_{Tj})G_{T2}(f_{Tk})C_jC_k \times \Big(\\
& \cos\left(2\pi f_{Tj}t + \beta_j + \phi_{S1}(f_{Sj}) + \phi_{T1}(f_{Tj}) - 2\pi f_{Tk}t - \beta_k - \phi_{S2}(f_{Sk}) - \phi_{T2}(f_{Tk})\right) \\
& -\cos\left(2\pi f_{Tj}t + \beta_j + \phi_{S1}(f_{Sj}) + \phi_{T1}(f_{Tj}) + 2\pi f_{Tk}t + \beta_k + \phi_{S2}(f_{Sk}) + \phi_{T2}(f_{Tk})\right) \\
& -\cos\left(2\pi f_{Tj}t + \beta_j + \phi_{S1}(f_{Sj}) + \phi_{T2}(f_{Tj}) - 2\pi f_{Tk}t - \beta_k - \phi_{S2}(f_{Sk}) - \phi_{T1}(f_{Tk})\right) \\
& +\cos\left(2\pi f_{Tj}t + \beta_j + \phi_{S1}(f_{Sj}) + \phi_{T2}(f_{Tj}) + 2\pi f_{Tk}t + \beta_k + \phi_{S2}(f_{Sk}) + \phi_{T1}(f_{Tk})\right)\Big)
\end{aligned}
\tag{55}
$$

so

$$
\begin{aligned}
(A_jB'_k - B_kA'_j) =& 0.5\, G_{S1}(f_{Sj})G_{S2}(f_{Sk})G_{T1}(f_{Tj})G_{T2}(f_{Tk})C_jC_k \times \Big(\\
& \cos\left(2\pi(f_{Tj} - f_{Tk})t + \beta_j - \beta_k + \phi_{S1}(f_{Sj}) - \phi_{S2}(f_{Sk}) + \phi_{T1}(f_{Tj}) - \phi_{T2}(f_{Tk})\right) \\
& -\cos\left(2\pi(f_{Tj} - f_{Tk})t + \beta_j - \beta_k + \phi_{S1}(f_{Sj}) - \phi_{S2}(f_{Sk}) + \phi_{T2}(f_{Tj}) - \phi_{T1}(f_{Tk})\right) \\
& +\cos\left(2\pi(f_{Tj} + f_{Tk})t + \beta_j + \beta_k + \phi_{S1}(f_{Sj}) + \phi_{S2}(f_{Sk}) + \phi_{T2}(f_{Tj}) + \phi_{T1}(f_{Tk})\right) \\
& -\cos\left(2\pi(f_{Tj} + f_{Tk})t + \beta_j + \beta_k + \phi_{S1}(f_{Sj}) + \phi_{S2}(f_{Sk}) + \phi_{T1}(f_{Tj}) + \phi_{T2}(f_{Tk})\right)\Big).
\end{aligned}
\tag{56}
$$

For pairs of components with the same temporal frequency, i.e $f_{Tj} = f_{Tk} = f_T$,

$$
\begin{aligned}
(A_jB'_k - B_kA'_j) =& 0.5\, G_{S1}(f_{Sj})G_{S2}(f_{Sk})G_{T1}(f_{Tj})G_{T2}(f_{Tk})C_jC_k \times \Big(\\
& \cos\left(\beta_j - \beta_k + \phi_{S1}(f_{Sj}) - \phi_{S2}(f_{Sk}) + \phi_{T1}(f_T) - \phi_{T2}(f_T)\right) \\
& -\cos\left(\beta_j - \beta_k + \phi_{S1}(f_{Sj}) - \phi_{S2}(f_{Sk}) + \phi_{T2}(f_T) - \phi_{T1}(f_T)\right) \\
& +\cos\left(2\pi(2f_T)t + \beta_j + \beta_k + \phi_{S1}(f_{Sj}) + \phi_{S2}(f_{Sk}) + \phi_{T2}(f_T) + \phi_{T1}(f_T)\right) \\
& -\cos\left(2\pi(2f_T)t + \beta_j + \beta_k + \phi_{S1}(f_{Sj}) + \phi_{S2}(f_{Sk}) + \phi_{T1}(f_T) + \phi_{T2}(f_T)\right)\Big) \\
=& 0.5\, G_{S1}(f_{Sj})G_{S2}(f_{Sk})G_{T1}(f_{Tj})G_{T2}(f_{Tk})C_jC_k \times \Big(\\
& \cos\left(\beta_j - \beta_k + \phi_{S1}(f_{Sj}) - \phi_{S2}(f_{Sk}) + \phi_{T1}(f_T) - \phi_{T2}(f_T)\right) \\
& -\cos\left(\beta_j - \beta_k + \phi_{S1}(f_{Sj}) - \phi_{S2}(f_{Sk}) + \phi_{T2}(f_T) - \phi_{T1}(f_T)\right)
\end{aligned}
\tag{57}
$$

which, using the trigonometric identity that $\cos(A) - \cos(B) \equiv 2\sin((B+A)/2)\sin((B-A)/2)$, can be simplified to

$$
\begin{aligned}
(A_jB'_k - B_kA'_j) =& G_{S1}(f_{Sj})G_{S2}(f_{Sk})G_{T1}(f_T)G_{T2}(f_T)C_jC_k \\
& \times \sin\left(\beta_j - \beta_k + \phi_{S1}(f_{Sj}) - \phi_{S2}(f_{Sk})\right)\sin\left(\phi_{T2}(f_T) - \phi_{T1}(f_T)\right).
\end{aligned}
\tag{58}
$$

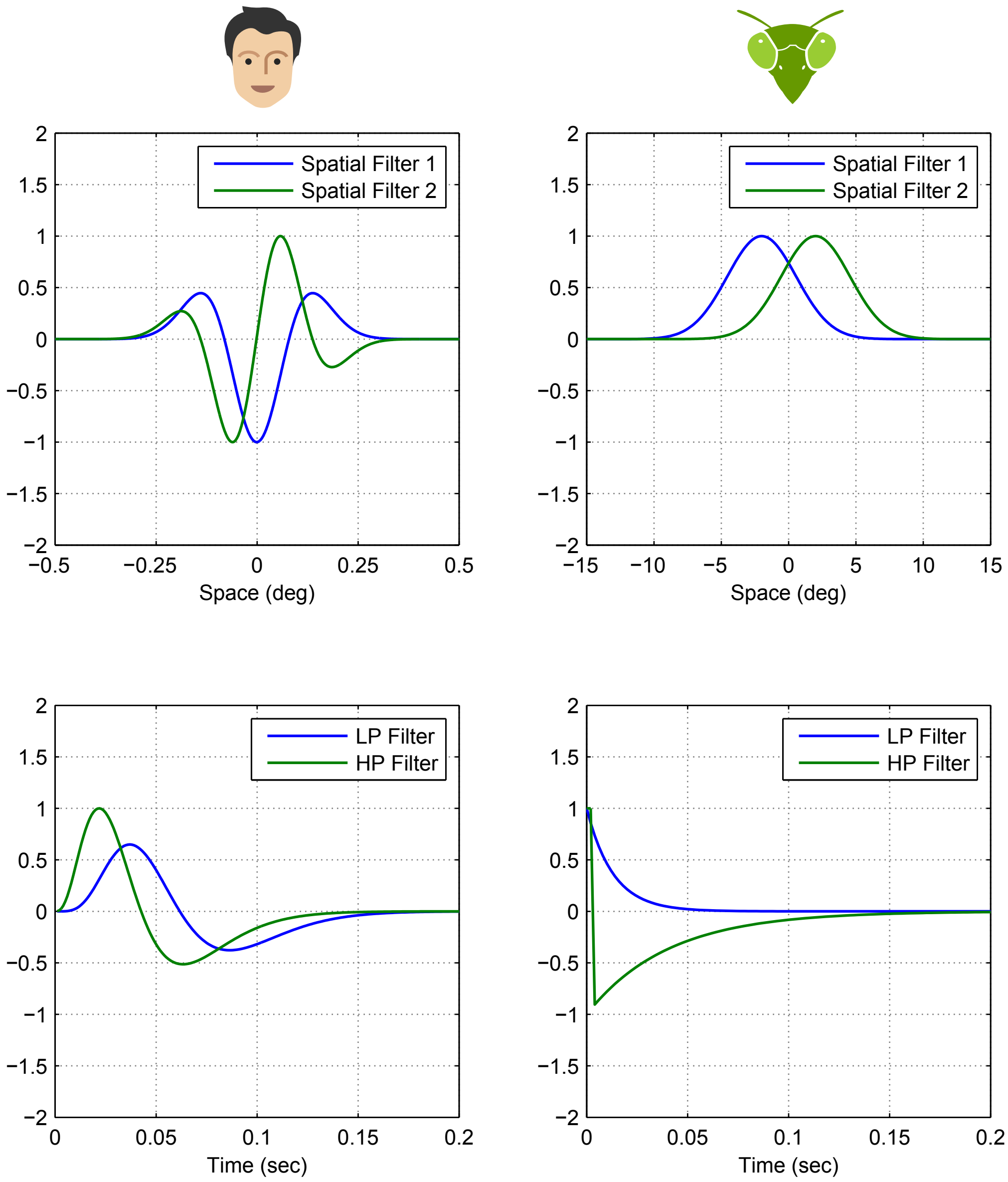

Figure 8. Impulse responses of spatial and temporal filters used in simulations
